# Radiographic Imaging Use Across Stages of Implant-Supported Rehabilitation: A Retrospective Single-Center Study

**DOI:** 10.3390/dj14090608

**Published:** 2026-09-19

**Authors:** Emanuel-Adrian Bratu, Ralph-Alexandru Erdelyi, Sergiu-Manuel Antonie, Ioan-Achim Borșanu, Remus Christian Bratu, Tiberiu Oancea, Ioan Sirbu

**Affiliations:** 1 Clinic of Implant Supported Restorations, “Victor Babeș” University of Medicine and Pharmacy Timisoara, 2 Eftimie Murgu Sq., 300041 Timisoara, Romania; ebratu@umft.ro (E.-A.B.); ioan.borsanu@umft.ro (I.-A.B.); remusbratuchristian@gmail.com (R.C.B.); todaysdentalhouston@gmail.com (T.O.); 2 Faculty of Electronics, Telecommunications and Information Technologies, Polytechnic University of Timisoara, 1 Mihai Viteazu Ave., 300222 Timisoara, Romania; 3 Discipline of Implanto-Prosthesis Therapy, Faculty of Dentistry, “Carol Davila” University of Medicine and Pharmacy, 37 Dionisie Lupu Street, District 2, 020021 Bucharest, Romania; ioan.sirbu@umfcd.ro

**Keywords:** CBCT, intraoral radiography, OPG, implantology, implant-supported restorations

## Abstract

**Background/Objectives:** Radiographic examinations are used at several stages of implant-supported rehabilitation, but patient-level utilization across an entire treatment pathway is not well described. This study mapped the use of panoramic radiography (OPG), cone-beam computed tomography (CBCT), and intraoral radiography across six analytical treatment stages in one clinical center. **Methods:** This retrospective single-center study included 50 consecutive adult patients with completed implant-supported rehabilitation and complete clinical and radiographic documentation from 2019 to 2025. Examination counts were summarized by modality and stage. Within-patient differences across stages were evaluated using the Friedman test. **Results:** The mean numbers of examinations per patient were 6.26 ± 3.11 for OPG, 2.72 ± 2.12 for CBCT, and 2.66 ± 3.43 for intraoral radiography. OPG was used throughout the pathway and was most frequent during prosthetic evaluation. CBCT was concentrated in the initial assessment and surgical stages, whereas intraoral radiography was concentrated during restorative and endodontic preparation and was not recorded during follow-up. **Conclusions:** The three imaging modalities showed different distributions across the retrospectively de-fined treatment stages. These findings describe imaging utilization in one institution and should not be interpreted as validation of a universal protocol or as evidence that the observed examination pattern was clinically optimal. Prospective multicenter studies incorporating examination-level indications, diagnostic yield, patient-specific dose, and clinical outcomes are required.

## 1. Introduction

Radiographic imaging complements clinical examination throughout im-plant-supported rehabilitation. Selection should address a defined diagnostic question, and established recommendations generally support two-dimensional imaging for initial assessment and CBCT when cross-sectional information is expected to influence patient management [1,2,3,4,5,6,7].

The modalities provide different information. Intraoral periapical radiography offers high spatial resolution for localized dental, peri-implant, and component-interface assessment, whereas panoramic radiography (OPG) provides a broad overview of both jaws and extensive rehabilitations but is limited by magnification, distortion, superimposition, and absence of buccolingual information [1,2,3,7,8,9]. CBCT depicts alveolar morphology and three-dimensional anatomical relationships and supports prosthetically driven or computer-assisted planning, but its higher and protocol-dependent exposure requires examination-level justification and patient-specific optimization [4,5,6,7,10,11,12,13,14,15,16,17].

After implant placement, standardized periapical radiography is generally appropriate for site-specific comparison of marginal bone levels and component interfaces. OPG may be useful when a broad overview is required, while CBCT should be reserved for specific questions that cannot be resolved through clinical examination and appropriately selected two-dimensional imaging [2,5,8,9,18,19,20].

Most recommendations address individual diagnostic questions or isolated treatment moments. Less is known about how different radiographic modalities are distributed across an entire implant-supported rehabilitation pathway in routine practice. Survey-based evidence has also indicated variability in the use of two-dimensional radiography and CBCT across clinical practice, highlighting the importance of examining imaging utilization within specific institutional settings [21]. Rather than proposing new radiographic indications or validating a clinical protocol, this retrospective study aimed to describe the patient-level distribution of OPG, CBCT, and intraoral radiography across six operationally defined treatment stages in one clinical center.

## 2. Materials and Methods

### 2.1. Study Design and Ethical Approval

This retrospective, single-center observational study included the radiographic records of 50 adult patients evaluated and treated between 2019 and 2025 in a private dental clinic as part of an implant-supported rehabilitation pathway. The six treatment stages were used as an operational framework for retrospective classification and not as a validated or prescriptive imaging protocol.

Eligible records were those of consecutive adult patients (18 years or older) evaluated and treated for implant-supported rehabilitation during the study period, with treatment completed and with complete clinical and radiographic documentation permitting examination counts to be assigned by imaging modality and analytical stage. Records were excluded if treatment had not been completed during the observation period, if essential radiographic documentation was missing, or if the available records did not permit reliable classification of an examination by modality or stage. All consecutive records meeting these criteria were included until the final cohort of 50 patients was obtained.

All consecutive eligible records were included. Because the study was retrospective, no prospective sample-size calculation was performed. The available cohort exceeded the minimum threshold of 40 patients established in the approved research protocol. A sensitivity analysis based on the noncentral chi-square approximation indicated that, with 50 patients, six repeated measurements, α = 0.05, and 80% power, the Friedman analysis could detect an overall Kendall’s W of approximately 0.07 or greater.

All patients included had provided written informed consent for the relevant stages of treatment and for the use of anonymized clinical and radiographic data for scientific and educational purposes. No additional imaging examination, intervention, or modification of patient management was introduced for research purposes.

The study was conducted in accordance with the Declaration of Helsinki and was approved by the Ethics Committee of Victor Babeș University of Medicine and Pharmacy Timișoara (approval no. 21, 8 January 2025). Clinical and radiographic data were anonymized before analysis and processed in accordance with institutional requirements for confidentiality and data protection.

### 2.2. Radiographic Equipment and Digital Workflow

The radiographic workflow included intraoral radiography, OPG, and CBCT. Decisions regarding modality selection were made by Emanuel-Adrian Bratu, Sergiu-Manuel Antonie, and Ioan-Achim Borșanu, three clinicians with formal training and substantial clinical experience in implantology and implant-prosthetic treatment planning. The overall planning process was coordinated by the senior implantologist, Emanuel-Adrian Bratu, who had more than 25 years of clinical and academic experience in oral implantology. Decisions considered the diagnostic requirements of the individual case, anatomical characteristics, available previous imaging, and the anticipated relevance of additional information for patient management.

At the investigated center, OPG was performed first to provide a two-dimensional overview of the dentition, jaws, existing pathology, and overall rehabilitation context. CBCT was subsequently prescribed for three-dimensional assessment of potential implant sites, including available bone dimensions and morphology and their relationships with relevant anatomical structures. The modalities were intended to address different diagnostic questions. Nevertheless, the retrospective database did not document whether OPG findings modified the subsequent indication for CBCT or whether both examinations in-dependently affected patient management. This sequence should therefore be interpreted as center-specific practice rather than as a universally recommended protocol.

**Figure 1:**
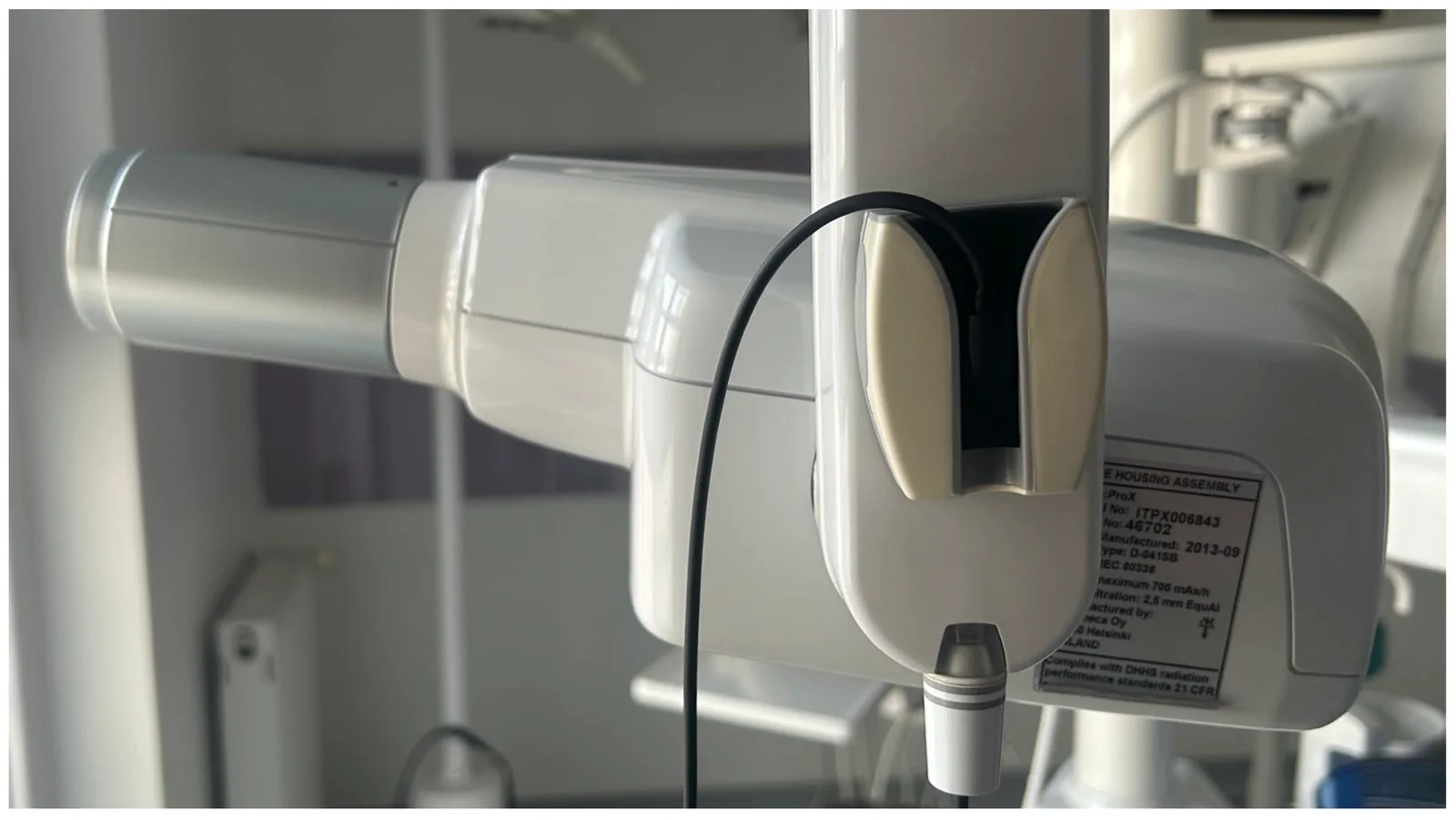
Planmeca ProX X-ray unit from the dental clinic.

Intraoral radiographs were acquired using a Planmeca ProX X-ray unit (Planmeca Oy, Helsinki, Finland) with digital image receptors and positioning devices (Figure 1). These examinations were used for localized assessment of the teeth and periapical tissues, evaluation of peri-implant marginal bone levels, and verification of implant-abutment and prosthetic-component seating.

**Figure 2:**
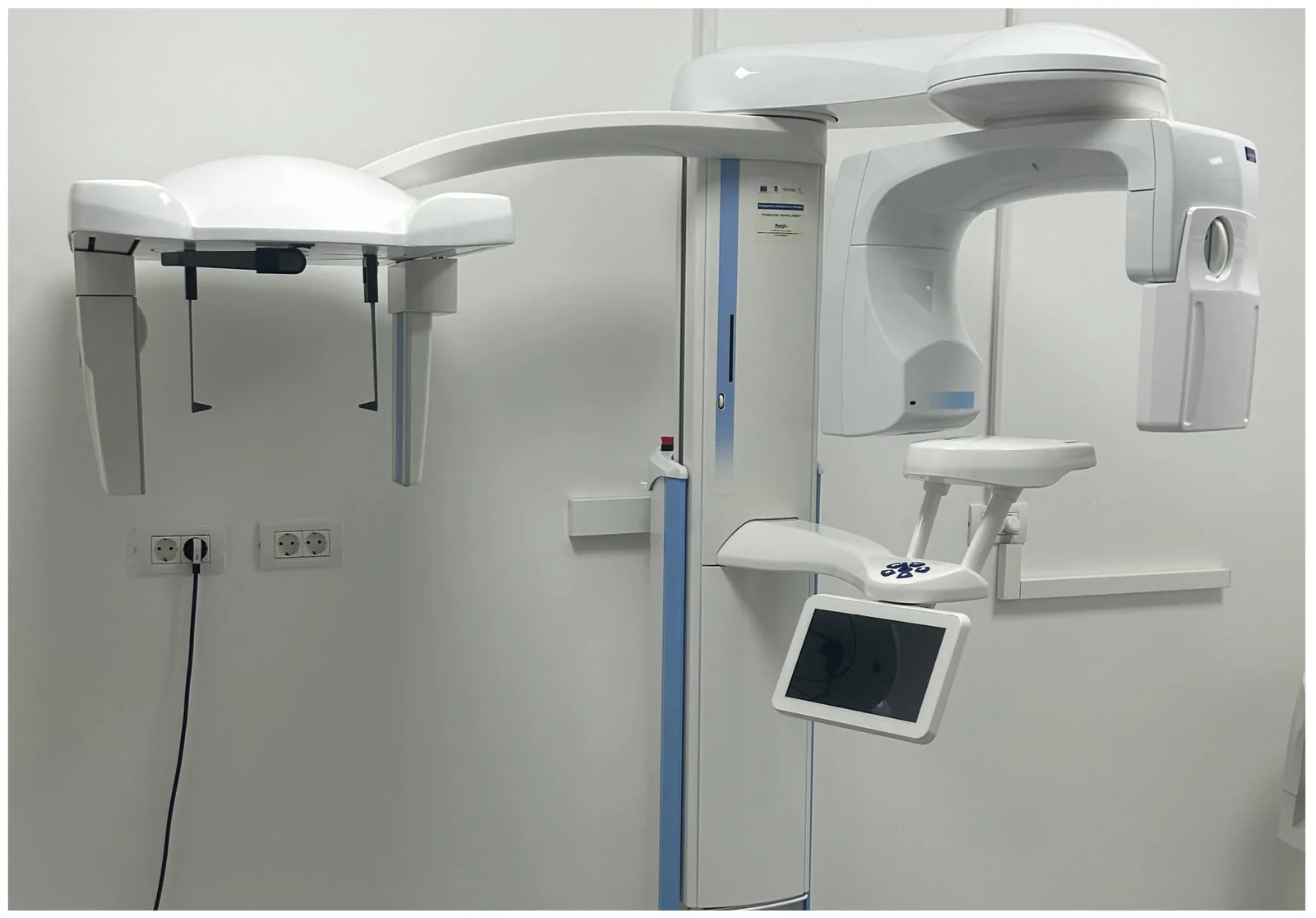
Planmeca ProMax X-ray unit from the dental clinic.

OPG examinations and CBCT examinations were acquired using a Planmeca ProMax Classic unit (Planmeca Oy, Helsinki, Finland) (Figure 2). Exposure parameters were adjusted individually according to patient anatomy, the region examined, and the diagnostic task. For CBCT examinations, the field of view and voxel size were selected according to the anatomical area and the level of detail required.

All radiographic examinations were reviewed and archived using Planmeca Romexis software 6.4.8 (Planmeca Oy, Helsinki, Finland). The software was used for image visualization, multiplanar reconstruction of CBCT datasets, cross-sectional assessment, linear measurements, and clinical documentation.

**Figure 3:**
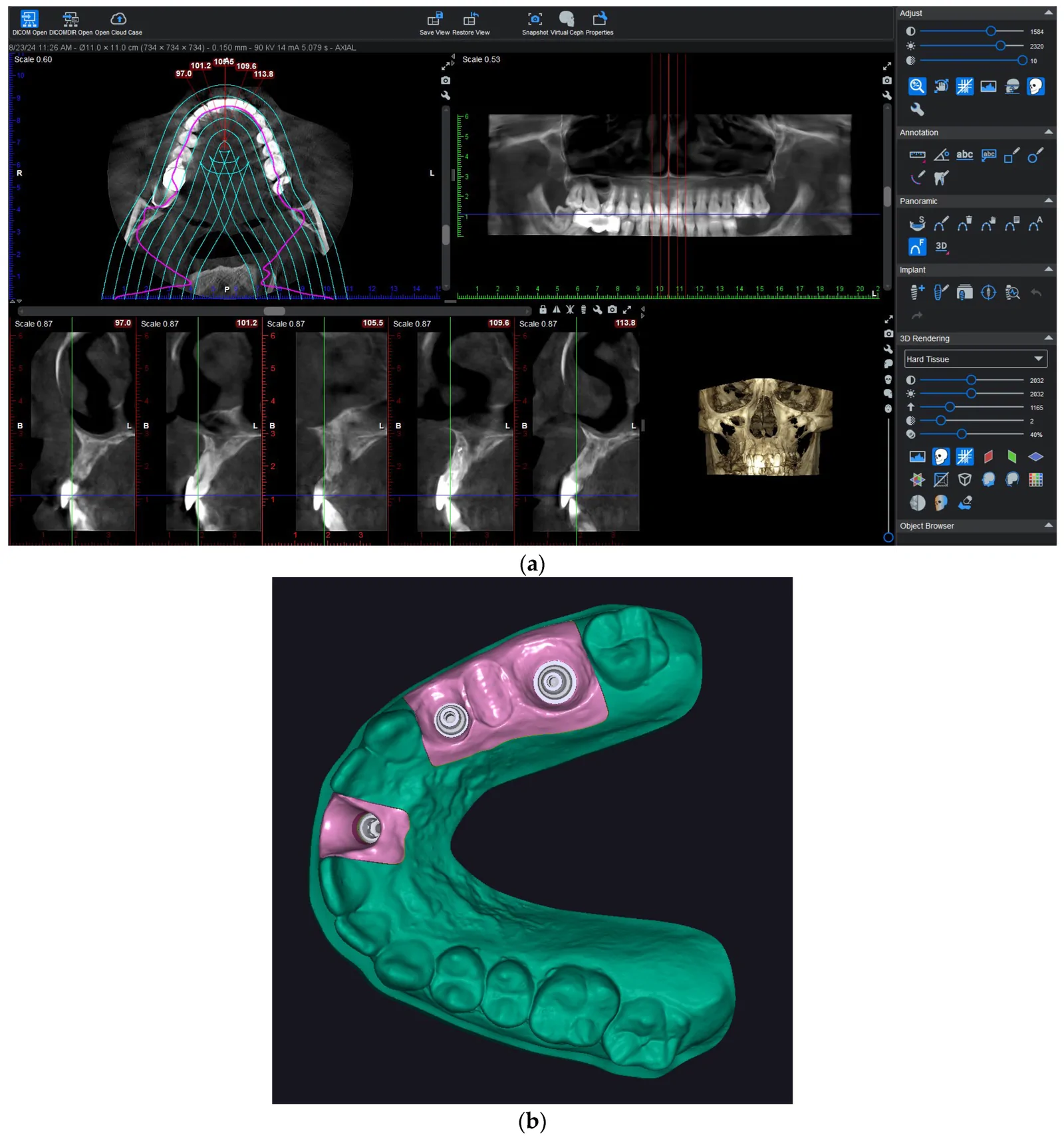
CBCT-based implant planning in Planmeca Romexis (**a**) and prosthetic surface design in Exocad (**b**).

In cases requiring prosthetically driven implant planning, CBCT data in Digital Imaging and Communications in Medicine (DICOM) format were integrated with surface data obtained by intraoral scanning and exported in Standard Tessellation Language (STL) format. Dataset alignment and virtual implant planning were performed using Exocad software 3.2 Elefsina (exocad GmbH, Darmstadt, Germany). This workflow enabled the proposed implant position to be evaluated in relation to the available bone, relevant anatomical structures, and planned prosthetic reconstruction (Figure 3).

### 2.3. Radiographic Modalities

The study evaluated three radiographic modalities: intraoral radiography, OPG, and CBCT. Each modality was selected according to the diagnostic task, anatomical region, and treatment stage.

#### 2.3.1. Intraoral Radiography

**Figure 4:**
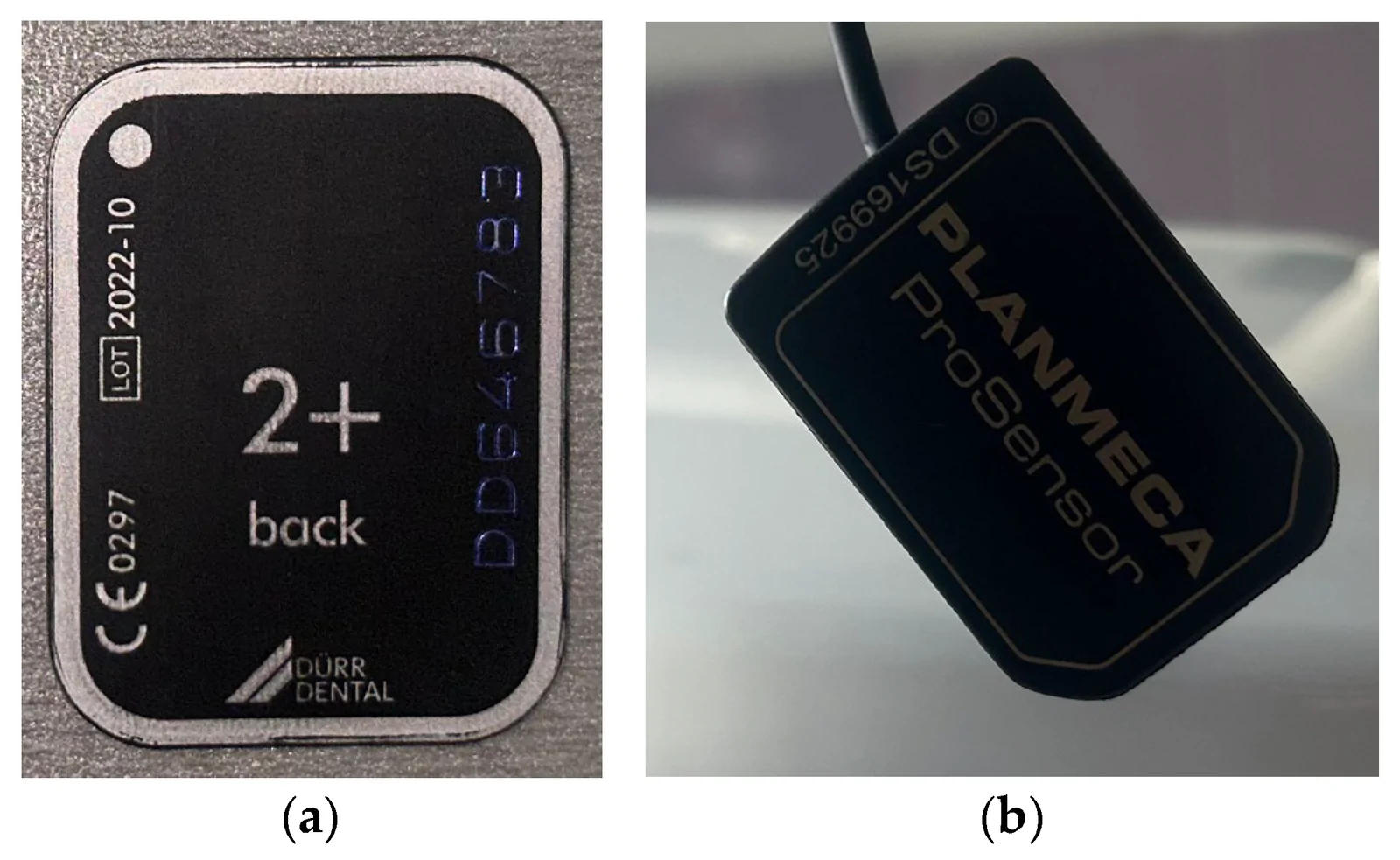
Intraoral image sensors available in the clinical workflow: (**a**) Photostimulable Phosphor Plate and (**b**) digital sensor.

Intraoral radiographs were acquired using the Planmeca ProX unit, which permits adjustment of tube voltage, tube current, and exposure time according to the anatomical region and patient characteristics. The system was compatible with both photostimulable phosphor (PSP) plates and direct digital sensors (Figure 4).

PSP plates provided greater flexibility during receptor positioning, particularly in patients with limited mouth opening or anatomical constraints. However, they required an additional scanning step before image visualization. Direct digital sensors provided immediate image acquisition but were more rigid and could be more difficult to position in posterior regions.

The radiographic examinations included in the study were predominantly acquired using the Planmeca ProSensor HD direct digital sensor (Planmeca Oy, Helsinki, Finland). Sensor size 2 was used for most adult patients. Positioning devices and the paralleling technique were employed whenever anatomical and clinical conditions permitted, particularly for examinations intended for longitudinal comparison.

**Figure 5:**
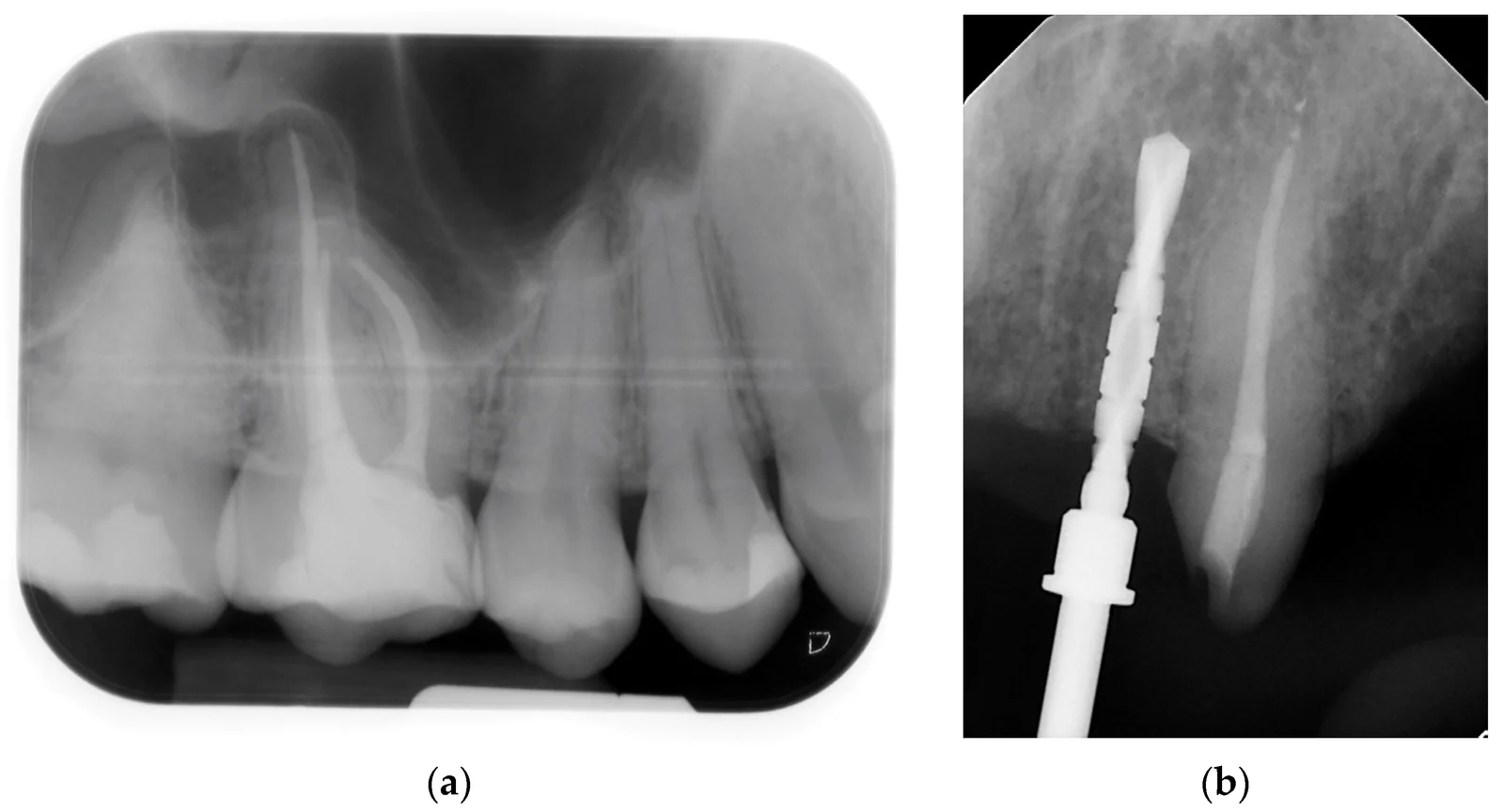
Illustrative examples of intraoral radiographs acquired using (**a**) a photostimulable phosphor plate (PSP) and (**b**) a direct digital sensor, showing the visual differences between the two receptor systems. No image-enhancement or noise-reduction filters were applied.

Intraoral radiography was used for localized evaluation of the periapical and periodontal status of retained teeth, assessment of endodontic treatment, intraoperative verification in selected cases, evaluation of peri-implant marginal bone levels, and confirmation of implant-abutment or prosthetic-component seating. Exposure parameters and beam collimation were adjusted to obtain diagnostically adequate images while limiting patient exposure. Illustrative examples acquired using the two receptor systems are presented in Figure 5.

The effective dose associated with an intraoral radiograph varies according to the receptor, beam collimation, exposure parameters, and anatomical region. Patient-specific doses were not available in the present retrospective study. Exposure settings were selected according to the diagnostic task and patient characteristics, in accordance with the principles of justification and optimization [16,17].

#### 2.3.2. OPG

Panoramic radiographs (orthopantomograms; OPGs) were acquired using the Planmeca ProMax Classic unit (Planmeca Oy, Helsinki, Finland). The system produces a two-dimensional image of the maxillofacial region through synchronized rotation of the X-ray source and image receptor around the patient.

The unit provides dedicated acquisition programs for adult and pediatric patients, edentulous arches, maxillary sinus evaluation, and temporomandibular joint imaging. The acquired images were transferred directly to Planmeca Romexis software, which allowed immediate visualization, image enhancement, diagnostic evaluation, and archiving.

During image acquisition, the patient was stabilized using the unit’s three-point positioning system. Light-beam guides were used to align the midsagittal plane, the anteroposterior position of the dental arches within the focal trough, and the occlusal reference plane. Patient-size selection and exposure parameters, including tube voltage and current, were adjusted according to patient morphology and the diagnostic requirements of the examination.

**Figure 6:**
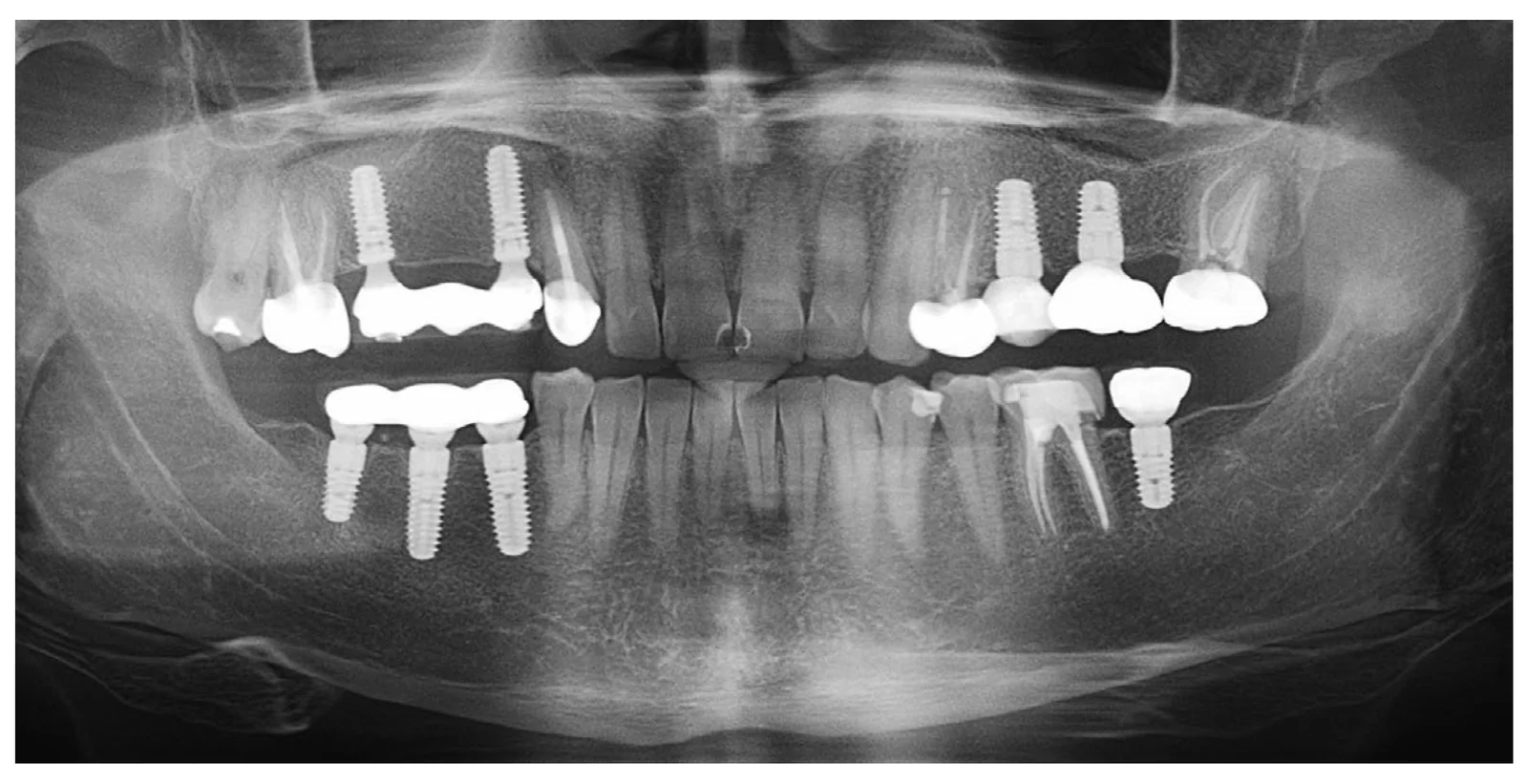
Representative panoramic radiograph acquired using the Planmeca ProMax system.

Digital OPG (Figure 6) provides lower spatial resolution than intraoral radiography. The image pixel size of the panoramic system was approximately 144–150 µm, depending on the selected acquisition and reconstruction settings, whereas intraoral imaging provided finer spatial detail. This difference, together with inherent image magnification and geometric distortion, limits the use of OPG for high-precision measurements. These limitations were considered when interpreting implant position, anatomical distances, and peri-implant bone levels.

The field of view of the standard adult panoramic program encompassed both dental arches and adjacent anatomical structures, including the mandibular canal, maxillary sinuses, and temporomandibular regions. This broad anatomical coverage allowed OPG to be used for initial assessment, postoperative documentation, evaluation of implant distribution in extensive rehabilitations, and selected follow-up examinations.

The effective dose associated with a standard adult OPG examination is generally within the range of approximately 10–15 µSv, although the actual value depends on the acquisition program, exposure parameters, patient size, and equipment configuration. Patient-specific dose indicators were not available for the present retrospective analysis; this contextual range should therefore not be interpreted as the dose received by individual participants.

#### 2.3.3. Cone-Beam Computed Tomography

Three-dimensional imaging was performed using the Planmeca ProMax Classic unit (Planmeca Oy, Helsinki, Finland), which supports CBCT acquisition with adjustable fields of view, voxel sizes, and exposure parameters. The system was used for volumetric reconstruction of dentoalveolar and craniofacial structures for diagnostic evaluation and implant planning.

CBCT examinations were acquired using fields of view adapted to the anatomical region and diagnostic task. The most frequently selected volumes were 8 × 5 cm and 11 × 8 cm. Voxel sizes ranging from 200 to 75 µm were available and were selected according to the level of anatomical detail required. Smaller voxel sizes were reserved for indications requiring finer assessment and were not used automatically, because increased spatial resolution may require higher exposure settings and generates larger datasets.

All CBCT datasets were reconstructed, evaluated, and archived using Planmeca Romexis software. Multiplanar reformations, cross-sectional images, panoramic reconstructions, and three-dimensional volume renderings were used according to the diagnostic question. In cases requiring static computer-assisted implant surgery or prosthetically driven planning, CBCT data in DICOM format were exported and merged with intraoral surface-scan data in STL format using Exocad software (exocad GmbH, Darmstadt, Germany).

**Figure 7:**
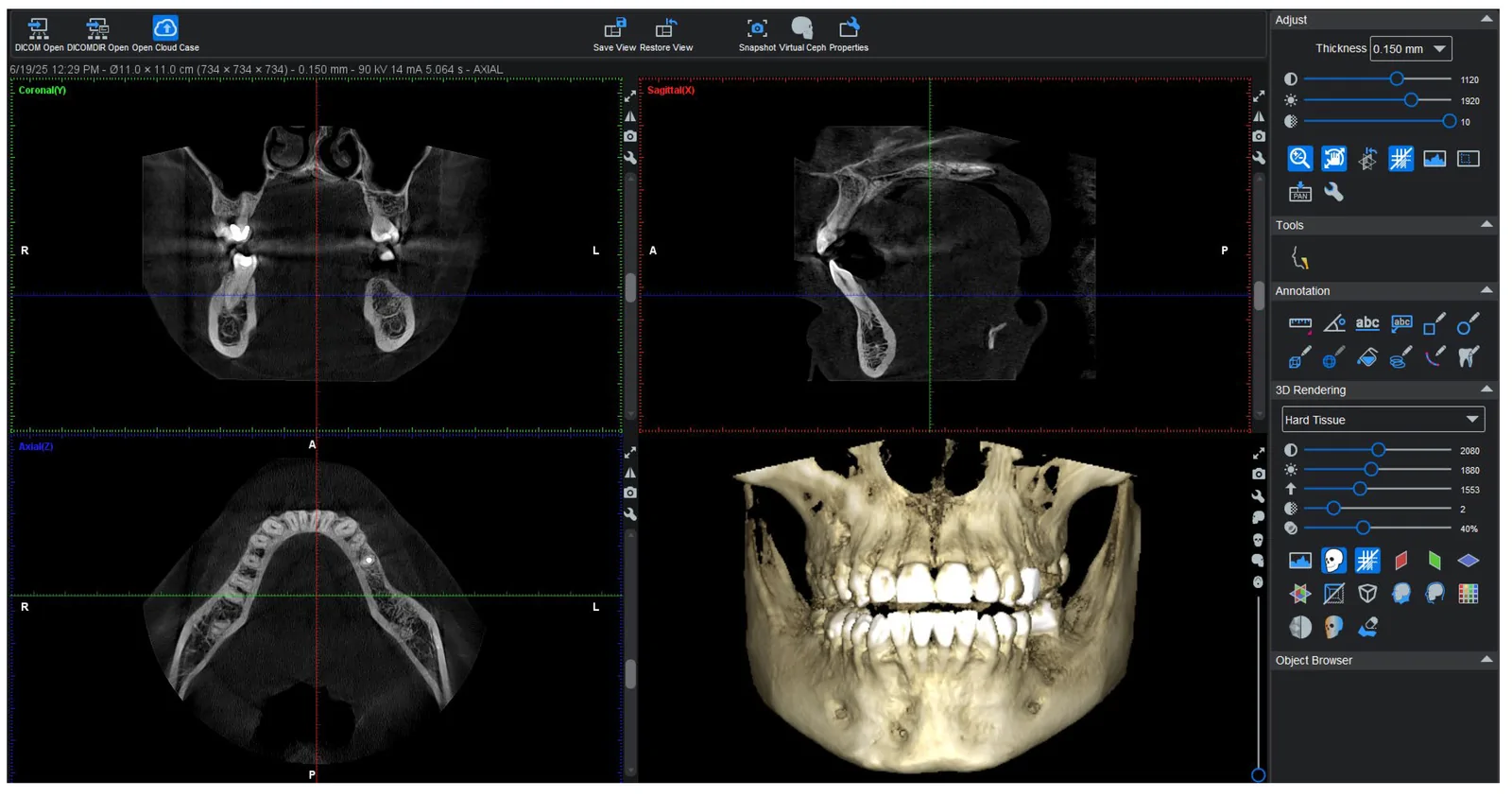
CBCT dataset displayed in Romexis 6.4.8 software using a field of view of 11 × 5 cm and a voxel size of 150 µm.

The principal applications of CBCT (Figure 7) within the clinical workflow were as follows:

- Three-dimensional assessment of alveolar bone volume and morphology before implant placement;
- Identification of relevant anatomical structures and potential risk areas;
- Planning of implant position, angulation, dimensions, and number;
- Postoperative assessment in selected full-arch or anatomically complex cases when two-dimensional imaging was insufficient;
- Evaluation of selected complications or suspected peri-implant bone defects that could not be characterized adequately using intraoral or panoramic radiography.

CBCT provided adequate spatial information for linear and angular measurements of mineralized structures but had limited value for direct soft-tissue assessment. Image quality was influenced by the selected voxel size, exposure parameters, patient movement, partial-volume effects, and artefacts generated by metallic implants or prosthetic components. When a removable screw-retained prosthesis produced diagnostically relevant artefacts, it was removed before acquisition if this was clinically feasible.

Radiation exposure varied according to field of view, voxel size, tube voltage, tube current, exposure time, patient size, and acquisition mode. The clinical protocols used with this system indicated contextual effective-dose estimates of approximately 35–85 µSv per examination; however, patient-specific dose indicators and complete acquisition parameters were not available for every examination. Restricted fields of view and low-er-dose modes were selected when they provided diagnostically adequate information for the clinical question [4,15,16,17,22].

### 2.4. Operational Classification of Treatment Stages

For retrospective coding, each examination was assigned to one of six operational stages corresponding to the temporal sequence documented in the investigated center. These categories were developed to organize existing records for analysis; they were not derived as an evidence-based classification, externally validated, or intended to prescribe when a radiographic examination should be performed.

The six analytical stages were: (1) initial diagnostic assessment; (2) restorative and endodontic preparation; (3) implant planning and intraoperative verification; (4) immediate postoperative assessment; (5) prosthetic evaluation; and (6) follow-up.

For each patient, OPG, CBCT, and intraoral examination counts were recorded separately within each analytical stage. The operational definitions used for classification are summarized in Table 1.

In the analyzed cohort, the initial assessment included one OPG and one CBCT ex-amination for each patient. The existing CBCT dataset was reused for planning when it remained representative of the clinical anatomy and provided adequate image quality and coverage. A repeat CBCT was not triggered solely by progression to another stage; it was considered when clinically relevant anatomical changes had occurred, the existing field of view or image quality was inadequate for the current task, or a new three-dimensional question was expected to influence patient management [2,5,6,23]. Because the database did not record the indication or diagnostic consequence of each repeat CBCT, the appropriateness of individual repeat examinations could not be determined.

Except for the center-specific initial sequence described above, examinations were not assigned automatically to a stage, and additional imaging was prescribed according to the requirements of the individual case. The analytical stages should not be interpreted as a prescriptive imaging schedule or universally applicable protocol.

### 2.5. Data Collection and Outcome Measures

The radiographic records of the 50 included patients were reviewed retrospectively. Patient identifiers were removed from the analytical dataset and replaced with numerical study codes.

For each patient, every examination was classified as OPG, CBCT, or intraoral radiography and assigned to one of the six operational stages. A value of zero indicated that the modality was not used during that stage and was not treated as missing data.

The primary outcome was the number of examinations performed with each imaging modality across the six treatment stages. Secondary outcomes included the total number of panoramic, CBCT, and intraoral examinations per patient and the relative distribution of the three modalities throughout the clinical pathway.

The study evaluated imaging utilization rather than individual exposure. Patient-specific dose indicators, complete acquisition parameters for every exposure, and cumulative radiation doses were not available and were therefore not study outcomes.

The dataset recorded examination frequency but not patient-specific dose indicators or complete acquisition parameters for every exposure. Consequently, cumulative radiation exposure could not be calculated, and examination count should not be used as a surrogate for radiation burden or optimization.

### 2.6. Statistical Analysis

Statistical analysis was performed using JASP 0.96.0 software (JASP Team, University of Amsterdam, Amsterdam, The Netherlands). Examination counts were summarized using the mean, sample standard deviation, median, interquartile range, minimum, and maximum. Sample standard deviations were calculated using (*n* − 1) in the denominator.

Because the same patients contributed discrete, non-normally distributed counts at each stage, a separate Friedman test was used for each modality. For each modality, this analysis addressed one limited question: whether examination counts within the same patients were distributed uniformly across the six operational stages. It was not designed to evaluate diagnostic accuracy, appropriateness, radiation optimization, or treatment outcome.

The magnitude of each overall stage effect was expressed using Kendall’s coefficient of concordance (W). When the Friedman test was significant, Conover pairwise comparisons with Holm adjustment were used. Stage 1 had no between-patient variability for OPG or CBCT, and Stage 6 had no intraoral examinations. These constant conditions generated extensive tied ranks and contributed to the omnibus statistics; the *p*-values therefore quantify non-uniformity but do not establish clinical appropriateness or protocol validity.

All statistical tests were two-sided. Adjusted *p*-values below 0.05 were considered statistically significant. Missing data were not imputed.

## 3. Results

### 3.1. Overall Imaging Utilization

The analysis included 50 patients with complete examination-count data. In total, 582 examinations were recorded: 313 OPG examinations (53.8%), 136 CBCT examinations (23.4%), and 133 intraoral radiographs (22.9%). All patients underwent at least one OPG and one CBCT examination, whereas intraoral radiography was recorded in 32 patients (64%).

**Figure 8:**
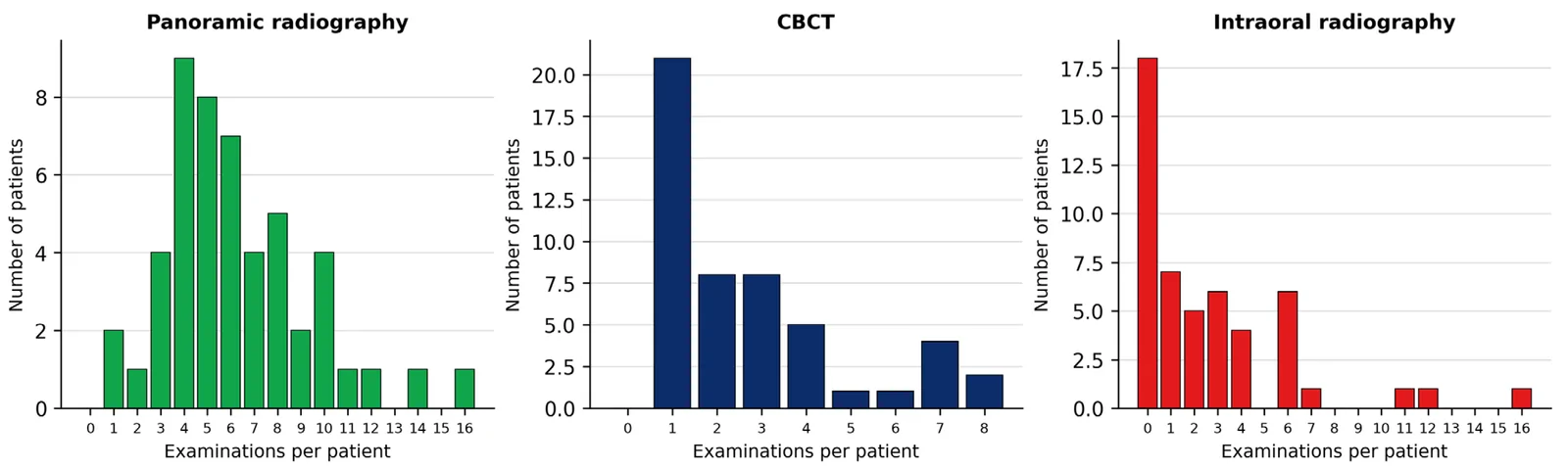
Distribution of the total number of radiographic examinations per patient according to imaging modality. Bars represent integer examination counts observed among the 50 patients.

Median examination counts per patient were 6.00 (IQR, 4.00) for OPG, 2.00 (IQR, 2.75) for CBCT, and 1.50 (IQR, 4.00) for intraoral radiography. Complete descriptive statistics are presented in Table 2 and the discrete distributions in Figure 8.

### 3.2. Stage-Specific Distribution of Imaging Modalities

**Figure 9:**
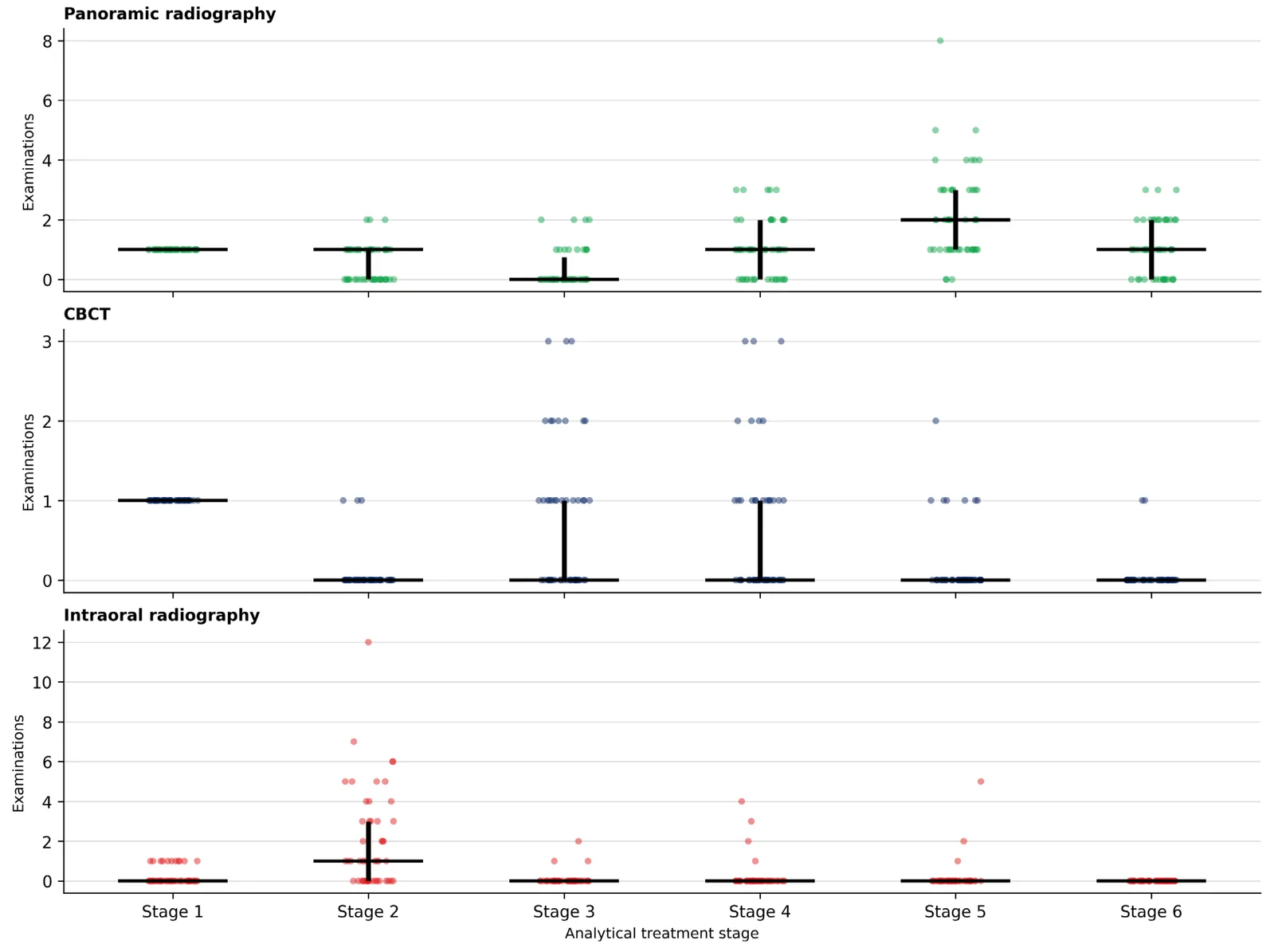
Patient-level distribution of radiographic examination counts according to imaging modality and treatment stage. Points represent individual patients; horizontal markers and error bars indicate the median and interquartile range.

Imaging utilization differed across analytical treatment stages. OPG was used throughout the pathway and was most frequent during prosthetic evaluation. CBCT was concentrated on the initial assessment and surgical stages and was uncommon during prosthetic treatment and follow-up. Intraoral radiography was concentrated during restorative and endodontic preparation and was not recorded during follow-up. Complete descriptive statistics are presented in Table 3 and Figure 9.

### 3.3. Proportion of Patients Examined at Each Treatment Stage

The proportion of patients undergoing at least one examination with each modality varied by stage (Table 4). At initial assessment, all patients underwent OPG and CBCT. OPG was recorded in 94% of patients during prosthetic evaluation and 68% during fol-low-up; CBCT was recorded in 48% during implant planning and 40% immediately post-operatively; intraoral radiography was recorded in 62% during restorative and endodontic preparation and in none during follow-up.

### 3.4. Comparisons Across Treatment Stages

Friedman tests indicated non-uniform within-patient examination count across the six analytical stages for OPG (Kendall’s W = 0.388), CBCT (W = 0.530), and intraoral radiography (W = 0.445; all *p* < 0.001; Table 5). Complete statistical outputs, including descriptive statistics, Friedman tests, Kendall’s W effect sizes, and Conover post hoc comparisons with Holm-adjusted *p*-values, are provided in Supplementary File S1.

These statistical differences should not be interpreted as evidence that the observed distributions were clinically optimal. Because the stages represented different activities and included structurally constant conditions, some modality-specific concentration was expected by design. The tests quantify within-patient stage-related patterns but do not establish appropriateness, diagnostic yield, radiation optimization, or improved outcomes.

## 4. Discussion

This retrospective analysis identified different distributions of OPG, CBCT, and intraoral radiography across six operational treatment stages in one institution. Its contribution is not the creation of new imaging indications or validation of a protocol, but patient-level mapping of examinations across a complete implant-supported rehabilitation pathway. The findings are therefore hypothesis-generating and primarily reflect the practice and experience of the investigated center.

All patients underwent OPG followed by CBCT at initial assessment. Within this center-specific sequence, OPG provided a broad two-dimensional overview, whereas CBCT addressed three-dimensional implant-site questions involving bone morphology and relevant anatomical relationships. However, the database did not record whether OPG findings altered the subsequent CBCT indication or whether each examination independently changed management. The sequence cannot therefore be recommended universally, and the justification of both examinations must remain patient- and question-specific [1,2,3,4,5,6,7].

Repeating CBCT requires scrutiny when a previous dataset is available. A new examination should not be triggered solely by progression to another stage but may be considered after clinically relevant anatomical change, when the existing field of view or image quality is inadequate, or when a new three-dimensional question is expected to influence management [2,5,6,23]. Because examination-level indications and diagnostic consequences were not recorded, the appropriateness of individual repeat CBCT examinations in this cohort could not be determined.

OPG provides a broad overview of both jaws and extensive implant-supported rehabilitations, whereas standardized intraoral radiography offers greater local detail for assessing implant-component interfaces and comparing mesial and distal marginal bone levels over time [9,24,25]. CBCT adds three-dimensional information for selected questions but should not be used routinely when clinical examination and appropriately selected two-dimensional imaging are sufficient [20,24]. The absence of intraoral radiographs during follow-up prevented standardized longitudinal assessment of peri-implant marginal bone levels and identifies a center-specific target for prospective audit rather than proof that another modality was inappropriate.

The Friedman results confirm a non-uniform distribution of counts within this dataset; they add no evidence that the observed pattern was clinically optimal or transferable to another institution. Stage 1 was constant for OPG and CBCT and Stage 6 was constant for intraoral radiography, producing tied ranks and contributing to the omnibus effects. Accordingly, medians, interquartile ranges, individual observations, and patient proportions are more informative for interpretation than *p*-values alone.

Radiation optimization cannot be inferred from examination counts alone. It requires examination-level justification and adaptation of acquisition parameters and image quality to the diagnostic task and individual patient [17,26]. Non-ionizing methods may provide complementary information for selected superficial dental structures. Optical coherence tomography generates high-resolution cross-sectional images without ionizing radiation and has been investigated as a complementary method for selected localized dental applications [27]. Its limited penetration depth prevents replacement of OPG or CBCT when alveolar bone, implant position, or deep anatomical relationships must be assessed.

Several limitations restrict interpretation. The retrospective single-center cohort included only 50 patients, and modality selection reflected the judgment of three experienced clinicians working in the same institution. The database lacked patient characteristics, implant and rehabilitation details, case complexity, examination-level indications, diagnostic yield, image-quality assessment, management changes, outcomes, and a comparison group. It also recorded frequency but not patient-specific dose indicators or complete acquisition parameters; cumulative exposure could not be calculated, and counts are not a surrogate for dose or optimization. Although sensitivity analysis indicated adequate power to detect the observed within-patient stage effects, statistical power does not establish representativeness or external validity.

Future prospective multicenter studies should record the indication, anatomical region, field of view, acquisition parameters, device-specific dose indicators, diagnostic findings, and management consequence of every examination. They should also distinguish rehabilitation types and include standardized periapical baseline and follow-up imaging when marginal bone change is an outcome. Whether the observed pattern increased or reduced cumulative exposure cannot be determined from the present data.

## 5. Conclusions

In this retrospective single-center cohort, OPG, CBCT, and intraoral radiography showed different distributions across six operationally defined treatment stages. OPG was used throughout the pathway, CBCT was concentrated in initial and surgical stages, and intraoral radiography was concentrated during restorative and endodontic preparation.

These findings describe imaging utilization in one institution and should be regarded as hypothesis-generating observations for prospective audit and multicenter research, not as validation of a stage-based protocol, evidence that the observed distribution was optimal, or a recommendation for universal practice. The small cohort, center-specific decision-making, absence of patient-level dosimetry, and lack of diagnostic-yield or outcome comparisons limit generalizability. Prospective multicenter studies are required to determine whether indication-based imaging pathways can reduce unnecessary examinations while maintaining sufficient diagnostic information.

## Figures and Tables

**Table 1:** Operational definitions used to assign radiographic examinations to the six analytical treatment stages.

Analytical Stage	Operational Context	Observed Modalities	Additional/Selective Use
1. Initial diagnostic assessment	General evaluation of the dentition, jaws, alveolar bone, and relevant anatomical structures	OPG followed by CBCT	Localized intraoral radiography
2. Pre-implant restorative and endodontic preparation	Evaluation and treatment of teeth retained within the planned rehabilitation	Intraoral radiography	Selected OPG or limited-FOV CBCT
3. Preoperative implant planning and intraoperative verification	Definitive implant planning and, when required, radiographic verification during surgery	CBCT	Selected OPG or intraoral radiography; repeat CBCT assessed against the available dataset
4. Immediate postoperative assessment	Documentation of implant position and relationship with adjacent structures	OPG and CBCT	Selected localized intraoral radiography
5. Prosthetic evaluation	Assessment and documentation of implant-supported prosthetic components	OPG	Selected intraoral radiography or CBCT
6. Follow-up	Radiographic overview after completion of prosthetic treatment	OPG	CBCT in two patients; no intraoral radiography

**Table 2:** Total number of radiographic examinations per patient according to imaging modality.

Imaging Modality	Total Examinations	Mean ± SD	Median (IQR)	Minimum–Maximum
OPG	313	6.26 ± 3.11	6.00 (4.00)	1–16
CBCT	136	2.72 ± 2.12	2.00 (2.75)	1–8
Intraoral radiography	133	2.66 ± 3.43	1.50 (4.00)	0–16

SD, standard deviation; IQR, interquartile range.

**Table 3:** Distribution of radiographic examinations according to analytical treatment stage and imaging modality.

Treatment Stage	OPG	CBCT	Intraoral Radiography
1. Initial diagnostic assessment	1.00 ± 0.00; 1.00 (0.00); 1–1	1.00 ± 0.00; 1.00 (0.00); 1–1	0.22 ± 0.42; 0.00 (0.00); 0–1
2. Restorative and endodontic preparation	0.58 ± 0.61; 1.00 (1.00); 0–2	0.06 ± 0.24; 0.00 (0.00); 0–1	1.94 ± 2.46; 1.00 (3.00); 0–12
3. Implant planning and intraoperative verification	0.34 ± 0.63; 0.00 (1.00); 0–2	0.74 ± 0.92; 0.00 (1.00); 0–3	0.08 ± 0.34; 0.00 (0.00); 0–2
4. Immediate postoperative assessment	1.06 ± 0.96; 1.00 (2.00); 0–3	0.60 ± 0.88; 0.00 (1.00); 0–3	0.20 ± 0.76; 0.00 (0.00); 0–4
5. Prosthetic evaluation	2.16 ± 1.52; 2.00 (2.00); 0–8	0.16 ± 0.42; 0.00 (0.00); 0–2	0.16 ± 0.77; 0.00 (0.00); 0–5
6. Follow-up	1.06 ± 0.91; 1.00 (2.00); 0–3	0.04 ± 0.20; 0.00 (0.00); 0–1	0.00 ± 0.00; 0.00 (0.00); 0–0

Values are presented as mean ± sample SD; median (IQR); minimum–maximum.

**Table 4:** Patients who underwent at least one radiographic examination, according to imaging modality and treatment stage.

Treatment Stage	Panoramic Radiography (%)	CBCT (%)	Intraoral Radiography (%)
1. Initial diagnostic assessment	50 (100%)	50 (100%)	11 (22%)
2. Restorative and endodontic preparation	26 (52%)	3 (6%)	31 (62%)
3. Implant planning and intraoperative verification	13 (26%)	24 (48%)	3 (6%)
4. Immediate postoperative assessment	34 (68%)	20 (40%)	4 (8%)
5. Prosthetic evaluation	47 (94%)	7 (14%)	3 (6%)
6. Follow-up	34 (68%)	2 (4%)	0 (0%)

Percentages were calculated using the complete cohort of 50 patients as the denominator. Patients could undergo more than one imaging modality during the same treatment stage.

**Table 5:** Friedman tests comparing imaging utilization across the six analytical treatment stages.

Imaging Modality	Friedman χ^2^	df	*p*-Value	Kendall’s W	Principal Post Hoc Pattern
OPG	96.92	5	<0.001	0.388	S5 > S1, S2, S3, S4, S6; S1, S4, S6 > S2, S3
CBCT	132.40	5	<0.001	0.530	S1 > S2–S6; S3, S4 > S2, S5, S6
Intraoral radiography	111.20	5	<0.001	0.445	S2 > S1, S3–S6; S1 > S6

Post hoc patterns are based on Conover pairwise comparisons with Holm adjustment. The “>” symbol indicates a significantly greater number of examinations.

## Data Availability

Additional information may be made available by the corresponding authors upon reasonable request, subject to applicable ethical and data-protection requirements.

## Supplementary Materials

The following supporting information can be downloaded at: https://www.mdpi.com/article/10.3390/dj14090608/s1, File S1: Supplementary statistical output containing the descriptive statistics, Friedman tests, Kendall’s W effect sizes, and Conover post-hoc comparisons with Holm-adjusted *p*-values reported in the manuscript.

## Abbreviations

The following abbreviations are used in this manuscript:

2D	Two-dimensional
3D	Three-dimensional
ALADAIP	As Low As Diagnostically Acceptable, Indication-oriented and Patient-specific
CBCT	Cone-beam computed tomography
DICOM	Digital Imaging and Communications in Medicine
FOV	Field of view
IQR	Interquartile range
OCT	Optical coherence tomography
OPG	Orthopantomogram
PSP	Photostimulable phosphor
SD	Standard deviation
STL	Standard Tessellation Language
