# Supplementary material for: Radiographic Imaging Use Across Stages of Implant-Supported Rehabilitation: A Retrospective Single-Center Study
Source: Dent J (Basel). 2026 Sep 19;14(9):608. doi: 10.3390/dj14090608 (PMC13605431; doi:10.3390/dj14090608)
Supplement: Supplementary file 1 [file dentistry-14-00608-s001.zip › dentistry-4495844-supplementary-File S1. Supplementary statistical output containing the descriptive statistics, Friedman tests, Kendall’s W effect sizes, and Conover post-hoc comparisons with Holm-adjusted p-v.pdf]

Results

Descriptive Statistics

Descriptive Statistics

	OPG	CBCT	RX INTRA
Valid	50	50	50
Missing	0	0	0
Median	6.000	2.000	1.500
Mean (arithmetic)	6.260	2.720	2.660
Std. Deviation	3.109	2.119	3.432
IQR	4.000	2.750	4.000
Minimum	1.000	1.000	0.000
Maximum	16.00	8.000	16.00

OPG examination counts across treatment stages

Friedman Test

Factor	X^2_F	df	p	Kendall's W
Phase	96.92	5	< .001	0.388

Conover Test

Conover's Post Hoc Comparisons - Phase

		T-Stat	df	W_i	W_j	r_{rb}	p	p_{Bonf}	p_{Holm}
Phase1	Phase2	4.278	245	190.0	133.5	0.778	< .001	< .001	< .001
	Phase3	6.322	245	190.0	106.5	0.805	< .001	< .001	< .001
	Phase4	0.530	245	190.0	183.0	−0.105	.597	1.000	1.000
	Phase5	5.111	245	190.0	257.5	−0.915	< .001	< .001	< .001
	Phase6	0.795	245	190.0	179.5	−0.091	.427	1.000	1.000
Phase2	Phase3	2.044	245	133.5	106.5	0.420	.042	.630	.168
	Phase4	3.748	245	133.5	183.0	−0.629	< .001	.003	.001
	Phase5	9.388	245	133.5	257.5	−0.977	< .001	< .001	< .001
	Phase6	3.483	245	133.5	179.5	−0.667	< .001	.009	.003
Phase3	Phase4	5.792	245	106.5	183.0	−0.818	< .001	< .001	< .001
	Phase5	11.43	245	106.5	257.5	−1.000	< .001	< .001	< .001
	Phase6	5.527	245	106.5	179.5	−0.818	< .001	< .001	< .001
Phase4	Phase5	5.641	245	183.0	257.5	−0.854	< .001	< .001	< .001
	Phase6	0.265	245	183.0	179.5	−0.024	.791	1.000	1.000
Phase5	Phase6	5.906	245	257.5	179.5	0.713	< .001	< .001	< .001

Note. Grouped by subject.

Note. Rank-biserial correlation based on individual signed-rank tests.

CBCT examination counts across treatment stages

Friedman Test

Factor	X^2_F	df	p	Kendall's W
Phase	132.4	5	< .001	0.530

Conover Test

Conover's Post Hoc Comparisons - Phase

		T-Stat	df	W_i	W_j	r_{rb}	p	p_{Bonf}	p_{Holm}
Phase1	Phase2	13.12	245	263.0	129.0	1.000	< .001	< .001	< .001
	Phase3	5.873	245	263.0	203.0	0.327	< .001	< .001	< .001
	Phase4	7.293	245	263.0	188.5	0.494	< .001	< .001	< .001
	Phase5	12.09	245	263.0	139.5	0.955	< .001	< .001	< .001
	Phase6	13.31	245	263.0	127.0	1.000	< .001	< .001	< .001
Phase2	Phase3	7.244	245	129.0	203.0	−1.000	< .001	< .001	< .001
	Phase4	5.824	245	129.0	188.5	−1.000	< .001	< .001	< .001
	Phase5	1.028	245	129.0	139.5	−0.556	.305	1.000	.667
	Phase6	0.196	245	129.0	127.0	0.200	.845	1.000	.845
Phase3	Phase4	1.419	245	203.0	188.5	0.375	.157	1.000	.628
	Phase5	6.216	245	203.0	139.5	0.938	< .001	< .001	< .001
	Phase6	7.439	245	203.0	127.0	0.948	< .001	< .001	< .001
Phase4	Phase5	4.796	245	188.5	139.5	1.000	< .001	< .001	< .001
	Phase6	6.020	245	188.5	127.0	0.931	< .001	< .001	< .001
Phase5	Phase6	1.224	245	139.5	127.0	0.750	.222	1.000	.667

Note. Grouped by subject.

Note. Rank-biserial correlation based on individual signed-rank tests.

RX Intra examination counts across treatment stages

Friedman Test

Factor	X^2_F	df	p	Kendall's W
Phase	111.2	5	< .001	0.445

Conover Test

Conover's Post Hoc Comparisons - Phase

		T-Stat	df	W_i	W_j	r_{rb}	p	p_{Bonf}	p_{Holm}
Phase1	Phase2	8.535	245	177.0	249.5	−1.000	< .001	< .001	< .001
	Phase3	2.354	245	177.0	157.0	0.636	.019	.290	.174
	Phase4	2.001	245	177.0	160.0	0.212	.046	.697	.325
	Phase5	2.296	245	177.0	157.5	0.319	.023	.338	.180
	Phase6	3.296	245	177.0	149.0	1.000	.001	.017	.011
Phase2	Phase3	10.89	245	249.5	157.0	1.000	< .001	< .001	< .001
	Phase4	10.54	245	249.5	160.0	0.909	< .001	< .001	< .001
	Phase5	10.83	245	249.5	157.5	1.000	< .001	< .001	< .001
	Phase6	11.83	245	249.5	149.0	1.000	< .001	< .001	< .001
Phase3	Phase4	0.353	245	157.0	160.0	−0.667	.724	1.000	1.000
	Phase5	0.059	245	157.0	157.5	−0.190	.953	1.000	1.000
	Phase6	0.942	245	157.0	149.0	1.000	.347	1.000	1.000
Phase4	Phase5	0.294	245	160.0	157.5	0.095	.769	1.000	1.000
	Phase6	1.295	245	160.0	149.0	1.000	.197	1.000	1.000
Phase5	Phase6	1.001	245	157.5	149.0	1.000	.318	1.000	1.000

Note. Grouped by subject.

Note. Rank-biserial correlation based on individual signed-rank tests.
